# Correction: Heuristic-enabled active machine learning: A case study of predicting essential developmental stage and immune response genes in *Drosophila melanogaster*

**DOI:** 10.1371/journal.pone.0305979

**Published:** 2024-06-18

**Authors:** Olufemi Tony Aromolaran, Itunuoluwa Isewon, Eunice Adedeji, Marcus Oswald, Ezekiel Adebiyi, Rainer Koenig, Jelili Oyelade

The second author’s name is spelled incorrectly. The correct name is: Itunuoluwa Isewon. The correct citation is: Aromolaran OT, Isewon I, Adedeji E, Oswald M, Adebiyi E, Koenig R, et al. (2023) Heuristic-enabled active machine learning: A case study of predicting essential developmental stage and immune response genes in *Drosophila melanogaster*. PLoS ONE 18(8): e0288023. https://doi.org/10.1371/journal.pone.0288023.
