# Supplementary material for: Heuristic-enabled active machine learning: A case study of predicting essential developmental stage and immune response genes in Drosophila melanogaster
Source: PLoS One. 2023 Aug 9;18(8):e0288023. doi: 10.1371/journal.pone.0288023 (PMC10411809; doi:10.1371/journal.pone.0288023)
Supplement: S1 Fig — A. HEAL results show a significantly lower false-positive rate except on the cancer dataset. B. UncAL produces higher true positives except on the Cancer and Credit-A data. C. RandAL performed well on the Credit-G data with a higher true positive and lower false negative. (DOCX) [file pone.0288023.s001.docx]

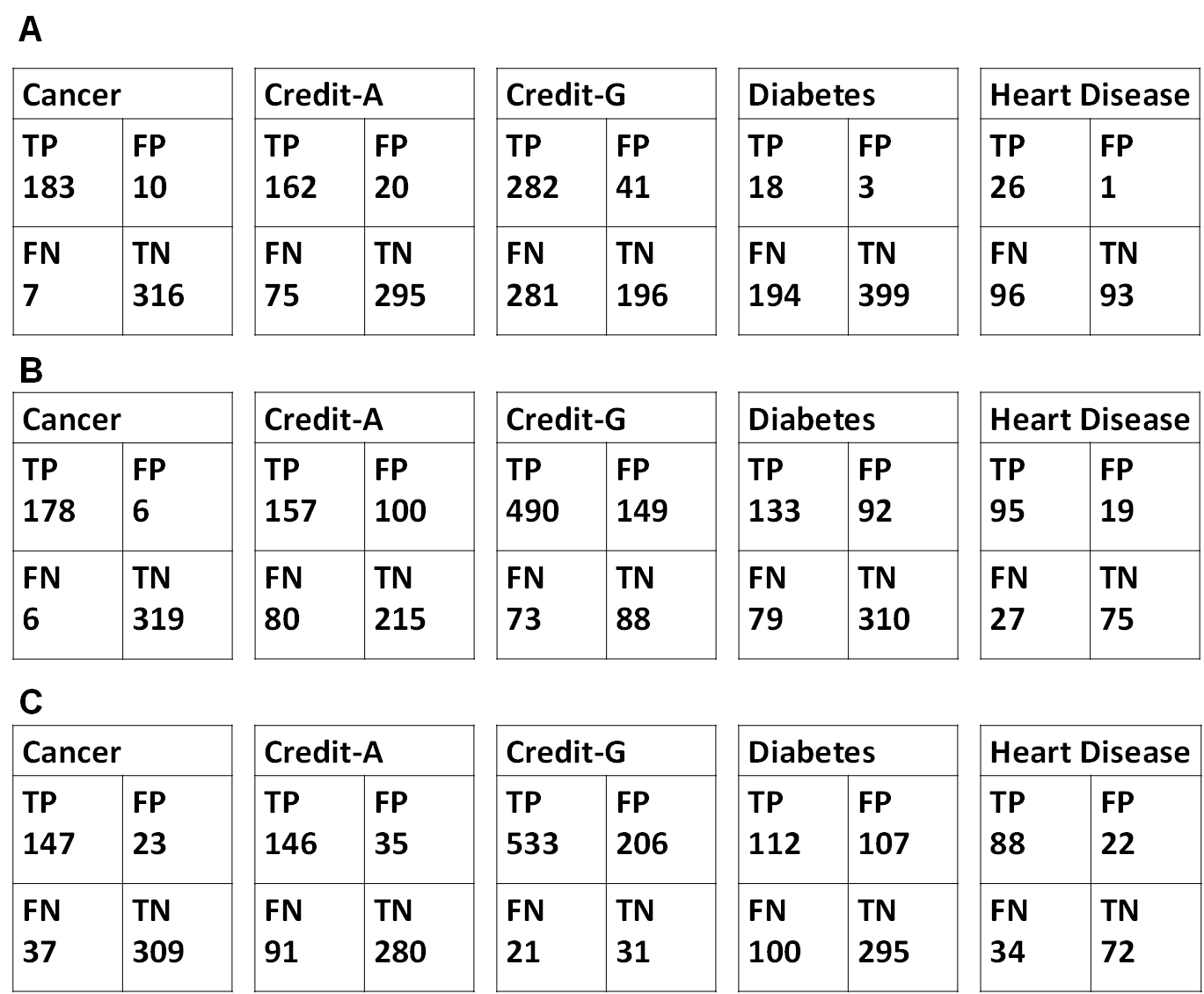


**Fig. S1:** Confusion matrix from the comparative analysis of HEAL and other techniques on five real-world datasets. A. HEAL results show a significantly lower false-positive rate except on the cancer dataset. B. UncAL produces higher true positives except on the Cancer and Credit-A data. C. RandAL performed well on the Credit-G data with a higher true positive and lower false negative.
